# The frequency and characterization of ovarian metastasis from nonovarian cancers using 18F-fluorodeoxyglucose PET/CT

**DOI:** 10.1093/bjro/tzaf004

**Published:** 2025-03-18

**Authors:** Nikoline D Frølich, Jeannette D Andersen, Helle D Zacho

**Affiliations:** Department of Nuclear Medicine and Clinical Cancer Research Center, Aalborg University Hospital, 9000 Aalborg, Denmark; Department of Nuclear Medicine and Clinical Cancer Research Center, Aalborg University Hospital, 9000 Aalborg, Denmark; Department of Nuclear Medicine and Clinical Cancer Research Center, Aalborg University Hospital, 9000 Aalborg, Denmark; Department of Clinical Medicine, Aalborg University, 9000 Aalborg, Denmark

**Keywords:** metastasis to the ovary, FDG PET/CT, ovarian mass

## Abstract

**Objective:**

Assessing the frequency of ovarian metastasis from nonovarian cancer (N-OC) and evaluate whether any PET-derived parameters can distinguish metastasis from primary ovarian cancer.

**Methods:**

Patients undergoing FDG PET/CT due to suspected ovarian malignancy from 2006 to 2021 with subsequent histologically proven ovarian metastasis from N-OC were included. Exclusion criteria included ovarian metastasis diagnosed prior to PET/CT or >3 months after. Baseline characteristics were collected from electronic medical records, and PET/CT data were analysed using Siemens syngo.via software.

**Results:**

Patients (*N* =1502) were scanned for suspected ovarian malignancies. Sixty-five patients (4%) were included. The most common origin of metastases was lower gastrointestinal cancer (*n* = 29, 45%), followed by gynaecological cancer (*n* = 10, 15%) and breast cancer (*n* = 9, 14%). Among patients with previous cancer history (*n* = 26), 18 experienced ovarian metastases from a known cancer. Time from primary diagnosis to ovarian metastasis ranged from 47 days to 11.4 years. There were no differences in maximized standardized uptake value, peak standardized uptake value, or clinical parameters between ovarian metastases and primary ovarian tumours.

**Conclusion:**

The frequency of ovarian metastases from N-OCs was 4%, the most common origin of metastases was lower gastrointestinal tract. Previous cancer history is an important factor in assessing an unknown tumour of the ovary, as metastases can occur several years later. No PET or clinical parameters were useful for separating primary ovarian cancer from ovarian metastases.

**Advances in knowledge:**

The study finds a low frequency of ovarian metastasis from N-OC and indicates that no PET or clinical parameters can distinguish ovarian metastasis from primary ovarian cancer.

## Introduction

Ovarian cancer accounts for 4% of all cancers in women and is the most lethal gynaecological malignancy worldwide in developed countries.^1^^,^^2^ In addition to primary ovarian cancers, the ovaries may also be affected by metastases originating from other malignant primary cancers, accounting for approximately 5%-20% of ovarian tumours.^2–4^

Distinguishing between primary ovarian cancer and metastases to the ovaries is important, as misinterpretation can result in suboptimal treatment, and patients with metastases to the ovaries have a worse survival rate than those with primary ovarian cancer (5-year survival rate of approximately 20% vs 40%).^5–7^

However, the differential diagnosis of metastases to the ovary vs primary ovarian cancer is difficult, as metastases frequently mimic the features of primary ovarian cancer, and histological examination is needed to determine the diagnosis.^2^^,^^3^^,^^6^

Conventional imaging modalities, including ultrasonography, CT, and MRI, are commonly utilized for the diagnosis of ovarian tumours. However, the imaging findings associated with ovarian metastases lack specificity and often overlap with those observed in primary ovarian cancer. Although various studies have reported on the radiological features of metastases to the ovaries, the delineation of distinct characteristics specific to this condition remains limited. Consequently, differentiating primary ovarian cancer from metastases to the ovaries poses a considerable diagnostic challenge.^8–12^

^18^F-fluorodeoxyglucose PET with CT (FDG PET/CT) is a well-established and valuable imaging modality with multiple applications, including initial staging, treatment response assessment, and restaging of various malignancies. In the context of ovarian cancer, FDG PET/CT plays a recognized role in differentiating between benign and malignant tumours, aiding in initial staging, and facilitating post-treatment follow-up. In patients who exhibit suspected malignant ovarian tumours based on conventional imaging or elevated serum tumour marker levels, the use of FDG PET/CT is warranted. However, limited information is available regarding FDG PET/CT findings of ovarian metastases.^8–11^

The maximized standardized uptake value (SUV_max_) has been investigated as a potential prognostic indicator in primary ovarian cancer,^13^ as well as for its clinical significance as a preoperative diagnostic tool to distinguish between benign, borderline, and malignant lesions.^9^ However, overlapping SUV values between these clinical entities have been shown.^10^ Whether the SUV_max_ may contribute to differentiating primary ovarian cancer from ovarian metastases remains understudied.

The aim of this study was to assess the frequency of metastasis to ovaries in nonovarian cancer patients. Additionally, we explored whether any characteristics, such as previous cancer history or FDG PET/CT findings, including SUV_max_, could distinguish patients with ovarian metastases from patients with primary ovarian cancer.

## Methods

### Patients

**Figure 1.**
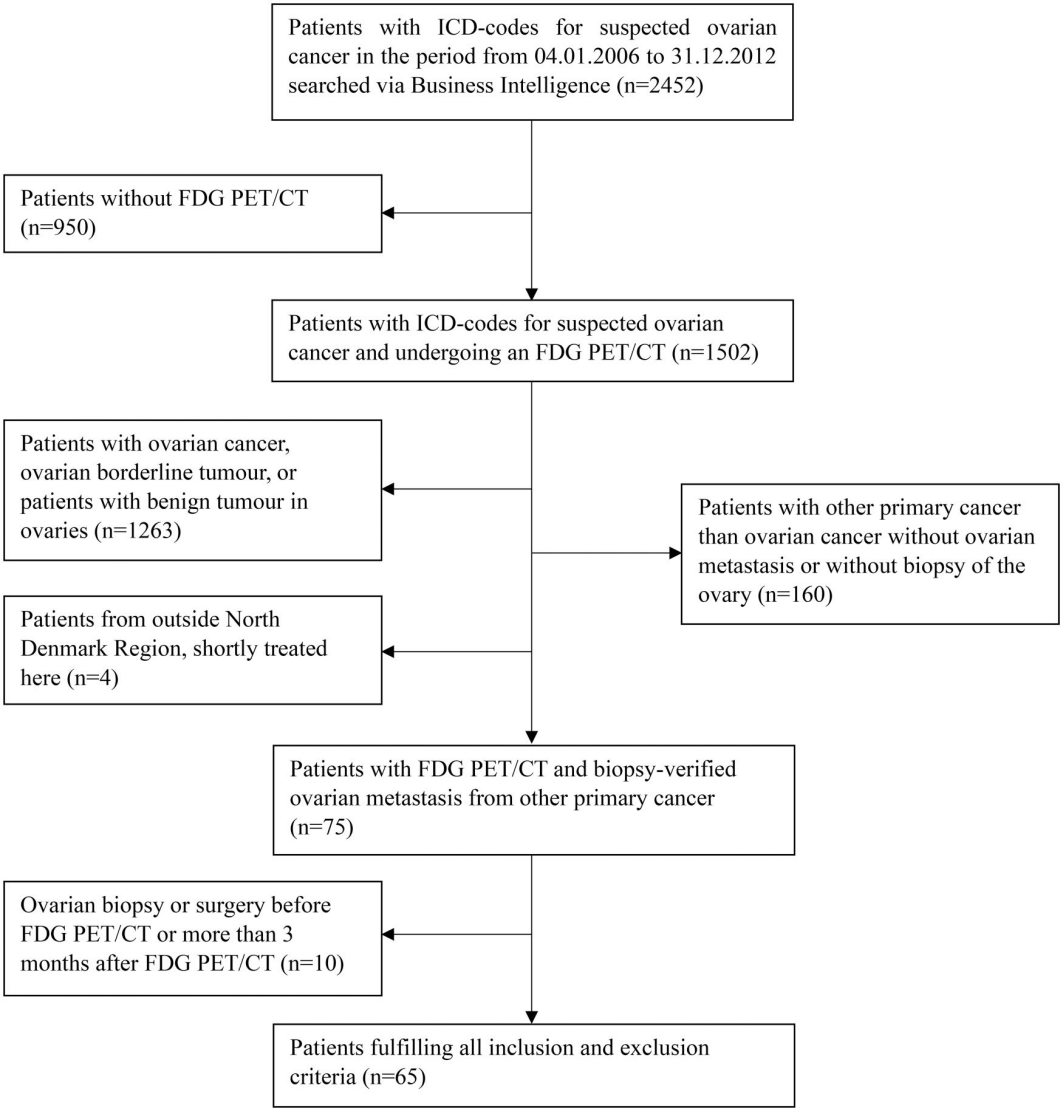
Flowchart specifying the inclusion of study patients. Abbreviations: FDG: ^18^F-fluorodeoxyglucose; ICD = International Classification of Diseases.

All patients who underwent FDG PET/CT from April 2006 to the end of December 2021 in the North Denmark Region due to suspicion of ovarian cancer were retrospectively evaluated and included in the study if the following criteria were met: (1) available history of biopsy or surgical specimen from the ovary/ovaries and (2) <3 months between the FDG PET/CT and biopsy and/or operation/removal of the ovaries. Patients were excluded if histological examination revealed benign disease of the ovary or if histological examination verified primary ovarian or fallopian tube cancer (borderline or malignant). The suspicion of ovarian cancer was based on clinical symptoms prompting referral to the Department of Gynaecology, and/or imaging either in terms of ultrasound or CT/MR, or prior imaging showing an ovarian mass/cyst or peritoneal carcinosis with no other obvious intraabdominal cancer. Finally, patients were excluded if the biopsy or operation/removal of the ovary was performed prior to the FDG PET/CT (Figure 1).

### Clinical data

Baseline characteristics, including age, weight and referring department, were collected from the patients’ electronic medical records. Any previous diagnosis of cancer and histological type was noted. The final diagnosis of ovarian metastases was based on pathology. Treatment of the cancer metastasizing to the ovaries was also noted. Treatment was divided into surgery, medical treatment, and combined treatment.

Cancer antigen 125 (CA-125) was noted if it was measured within 3 months before to 2 weeks after the FDG PET/CT. The risk of malignancy index (RMI) was calculated according to international and national guidelines.^14–16^ The data were collected retrospectively in June 2023.

### ^18^F-fluorodeoxyglucose PET/CT

From 2006 to 2017, images were acquired using a Discovery VCT scanner (GE Healthcare, Waukesha, Wisconsin, USA) using a fixed dose of 370 MBq, and after 2017, FDG PET/CT images were acquired on a Siemens Biograph mCT Flow 64 PET/CT system (Siemens Medical Solutions, Erlangen Germany) using 3.5 MBq/kg bodyweight (maximal dose of 400) according to EANM guidelines.^17^ All patients fasted for 6 h prior to the FDG injection. Blood glucose levels were measured in all patients, and patients with blood glucose levels above 11.0 mmol/L were cancelled and given a new appointment after regulation of the blood glucose. The time from injection to scan was aimed at 60±10 min.^18^

In general, FDG PET/CT was conducted with contrast-enhanced high-dose CT unless the patient had a known allergy to CT contrast or if a contrast-enhanced CT had been performed within one month prior to PET/CT. In such cases, a low-dose nonenhanced CT was acquired.

### Imaging analysis

Every FDG PET/CT scan was performed as part of daily clinical practice and evaluated by at least 2 observers, with at least 1 observer with extensive expertise in ovarian cancer. Contrast-enhanced CTs were assessed by a radiologist. However, FDG PET/CTs with low-dose CT could be assessed by 2 nuclear medicine physicians or 1 nuclear medicine physician and 1 radiologist.

The FDG PET/CT was evaluated based on the guidelines available at the time of the scan and with knowledge regarding variants and pitfalls in the evaluation of potential ovarian cancer.^19^^,^^20^ The original evaluations of the FDG PET/CT scans were divided into 4 groups depending on the original conclusion from the scan evaluation: (1) no signs of malignancy, including benign-looking ovaries; (2) suspected malignant ovarian tumour(s); (3) suspected nonovarian primary cancer with metastases to the ovary(es); and (4) suspected malignant ovarian tumour with a synchronous nonovarian malignant tumour.

For the retrospective analyses of semiquantitative measures, the FDG PET/CT scans were retrieved and reviewed using the Siemens syngo.via workstation (Siemens, Erlangen, Germany). The mean standardized uptake value (SUV_mean_) of the liver and aorta as well as the SUV_max_ and peak standardized uptake value (SUV_peak_) of the ovarian metastasis were recorded. In the case of bilateral metastasis, the lesion with the highest value was recorded.

### Statistics

Descriptive statistics are shown as the mean for normally distributed data and as the median when the data were not normally distributed. All the statistical analyses were carried out using Stata 17 (StataCorp. 2023. Stata Statistical Software: Release 17; StataCorp LLC, College Station, TX).

## Results

During the period from 2006 to 2021, 2452 patients were identified with International Classification of Diseases (ICD) codes for confirmed or suspected ovarian neoplasms, and 1502 patients underwent FDG PET/CT scans as part of the primary workup, of whom 65 patients (65/1502, 4%) were included in this study due to histology-proven metastases in the ovary(ies) from nonovarian cancer (Figure 1). The mean age at the time of FDG PET/CT was 59.4 years (range 32-89). The patients’ baseline characteristics and referring department are listed in Table 1. Most of the patients were referred for an FDG PET/CT scan from the Department of Gynaecology (75%), followed by the Department of Gastrointestinal Surgery (11%).

**Table 1.** Patient demographics (*n* = 65).

		Range
Age at scan (years), mean	59.4	32-89
Weight (kg), mean	72.6	48-130
Tracer (MBq), median	367	179-419
Glucose (mmol/L),^a^ median	5.5	4.2-8.6
Time from tracer injection to scan (min),^a^ median	63	53-110
Known previous cancer, n (%)	26 (40%)	
Referring department, n (%)		
Department of Gynecology	49 (75%)	
Department of Gastrointestinal Surgery	7 (11%)	
Department of Oncology	5 (8%)	
Other	4 (6%)	

a Available for *n* = 38.

The most common origin of the ovarian metastases was lower gastrointestinal cancer (*n* = 29, 45%), followed by nonovarian gynaecological cancers (*n* = 10, 15%) and breast cancer (*n* = 9, 14%) (Table 2). Prior to the suspicion of an ovarian tumour, 26 patients (40%) had a known diagnosis of nonovarian cancer; the majority of these patients had breast cancer (*n* = 8, 31%), lower gastrointestinal cancer or nonmelanoma skin cancer (both *n* = 6, 24%), and 6 patients had 2 or more previous cancer diagnoses (Table 3). Among the 26 patients with known nonovarian cancer, metastases in the ovary(ies) originated from known cancer in 18 patients (69%). The median time from the diagnosis of known nonovarian cancer to the development of ovarian metastasis was 4.3 years, ranging from 47 days to 11.4 years. In patients with lower gastrointestinal tract cancer (*n* = 7), the mean duration was 2.3 years, and in patients with breast cancer (*n* = 7), the mean duration was 7 years. Two patients had upper gastrointestinal cancer with 0.9 and 4.4 years to metastasis, respectively, and 2 patients had cancer in the urinary tract with 5 and 5.1 years to ovarian metastasis.

**Table 2.** Origin of ovarian metastases and the SUV_max_ and SUV_peak_ values.

Origin of ovarian metastases	*N* (%)	SUV_max_, mean (range)	SUV_peak_, mean (range)
Lower gastrointestinal	29 (44.6)	4.96 (1.28-13.74)	3.69 (0.99-11.23)
Gynaecologic	10 (15.4)	7.10 (0.75-26.69)	5.09 (0.65-15.86)
Breast	9 (13.9)	6.86 (1.84-14.85)	5.66 (1.55-12.50)
Upper gastrointestinal	6 (9.2)	4.10 (2.45-5.57)	2.83 (1.86-4.08)
Pancreas/bile duct	6 (9.2)	4.33 (2.27-6.26)	3.35 (1.61-5.34)
Urinary tract	3 (4.6)	8.03 (2.70-10.81)	6.41 (2.07-8.77)
Haematologic	2 (3.1)	1.83 (1.09-2.57)	1.45 (0.99-1.91)

**Table 3.** Origin of previous cancer (*n* = 26) and how many resulted in the ovarian metastasis per group (*n* = 18).

Origin of previous cancer	*N* (%)	Resulted in ovarian metastasis
Upper gastrointestinal	1 (3.9)	1
Lower gastrointestinal	6 (23.1)	6
Breast	8 (30.8)	7
Urinary tract	1 (3.9)	1
Skin (nonmelanoma)	6 (24.1)	0
Thyroid	1 (3.9)	0
Several cancers with more than one origin^a^	3 (11.5)	3^b^

a One patient with both breast cancer and cancer in the urinary tract, one patient with 2 types of lymphoma and upper gastrointestinal cancer and one patient with nonmelanoma skin cancer and lower gastrointestinal cancer.

b Origin of tumour metastasizing to the ovary was respectively cancer from the urinary tract, upper gastrointestinal, and lower gastrointestinal cancer.

CA-125 levels were available for 59 patients and were elevated in 49 patients (>35 kU/L). The median CA-125 concentration was 123 kU/L (range 12-7000 kU/L). Based on the CA-125 level and ultrasonography, 28 patients had a calculated RMI recorded in their medical journal. In 6 patients, the recorded RMI score was >200, but this was not further specified. In the remaining 22 patients, the median RMI was 590.

### ^18^F-fluorodeoxyglucose PET/CT

**Figure 2.**
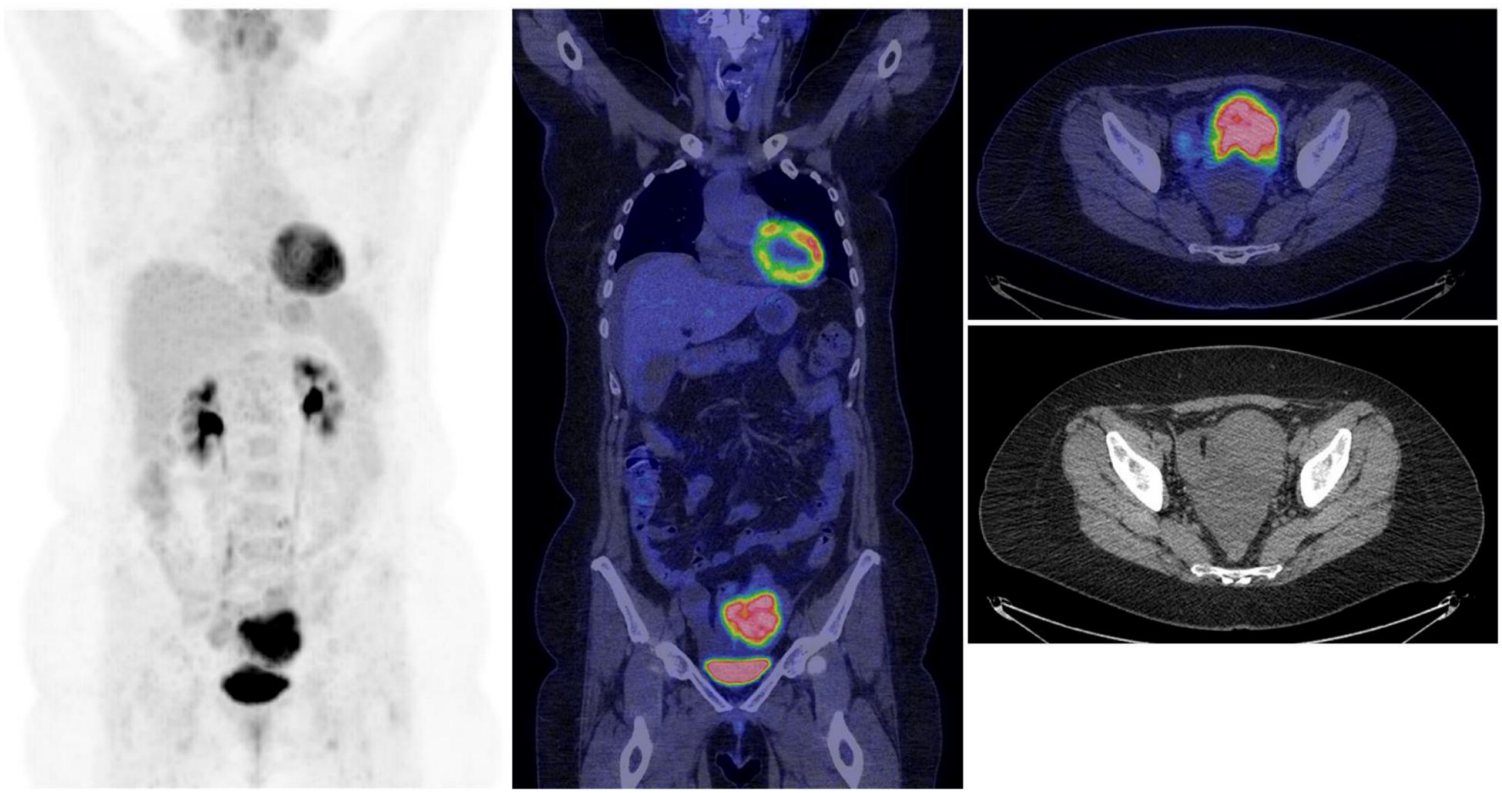
A 55-year-old woman was admitted with acute abdominal pain and examined via a CT scan showing an enlarged ovary; thereafter, the patient was referred for an ^18^F-fluorodeoxyglucose PET CT (FDG PET/CT) scan. The FDG PET/CT scan revealed high FDG-uptake in a suspected primary ovarian tumour originating from the left ovary with both solid and cystic components with no evidence of disseminated disease. The patient had previously been treated for breast cancer by lumpectomy and axillary lymph node dissection, followed by chemotherapy, radiation therapy and anti-estrogen therapy six and a half years prior to the current FDG PET/CT and the pathological diagnosis of the ovarian tumour was metastasis from the previous breast cancer. The SUV_max_ of the metastasis was 14.3.

**Figure 3.**
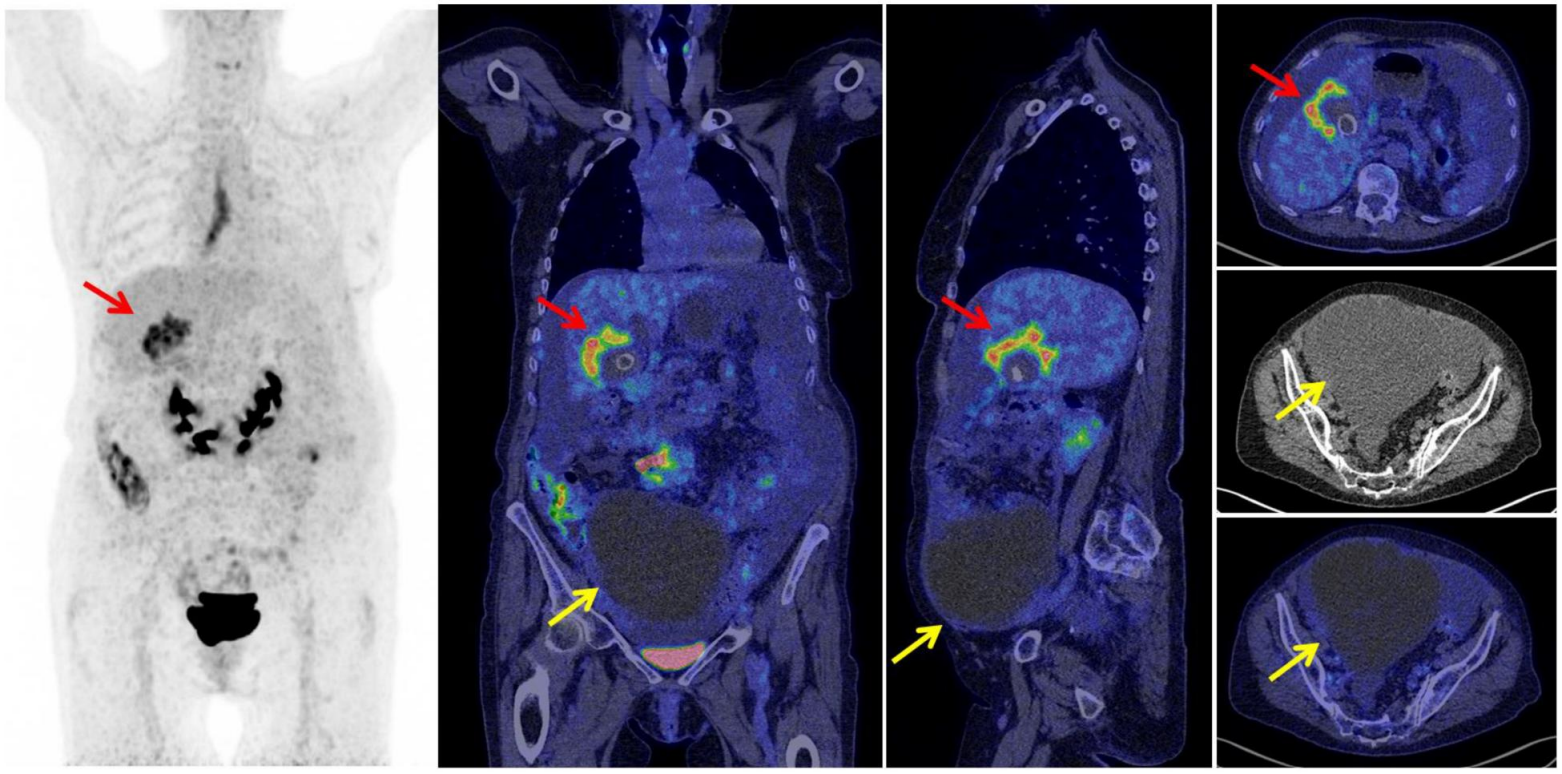
A 73-year-old woman was referred by her general practitioner due to weight loss and abdominal pain. She was examined via ultrasonography, which revealed a multichambered cyst in the pelvis measuring 20×11 cm. She was referred to the Department of Gynaecology, where she underwent an ^18^F-fluorodeoxyglucose PET CT (FDG PET/CT) scan. FDG PET/CT revealed a primary tumour located in the gallbladder (red arrow) with cystic metastasis to the ovary (yellow arrow). The SUV_max_ of the ovarian metastasis was 2.27.

Based on the original written reports from the FDG PET/CT scan evaluations, more than half of the FDG PET/CT evaluations suggested that the scan represented primary ovarian cancer (*n* = 37, 57%) (Figure 2), whereas in 8 patients (12%), the FDG PET/CT evaluation suggested primary ovarian cancer and synchronous nonovarian cancer. In 11 patients (17%), the correct diagnosis of nonovarian cancer with metastases to the ovary(ies) was suggested (Figure 3). Finally, FDG PET/CT did not reveal any signs of malignant disease in the ovaries in 9 patients (14%).

The mean SUV_max_ of the ovarian metastases was 5.39 (range 0.75-26.69). The mean SUV_mean_ of the aorta was 1.48, and the mean SUV_mean_ of the liver was 2.02.

The SUV values for the ovarian metastases based on the origin of the primary cancer are listed in Table 2. The highest SUVs were found in the ovarian metastases from gynaecological, breast and urinary tract cancers (SUV_max_ from 6.8 to 8.0), whereas the lowest SUV was detected in the ovarian metastases originating from haematologic cancer (SUV_max_ 1.8), but the values of the individual primary cancer types overlapped.

### Follow-up

Patients were followed for a median of 8.8 years. During follow-up, 51 of 65 patients (78%) died, and the mean time from FDG PET/CT to death was 2.1 years, ranging from 26 days to 10 years.

After being diagnosed with ovarian metastases, 21 (32%) patients underwent surgery, 42 (65%) underwent surgery combined with either chemotherapy or radiation, 1 patient (1.5%) received chemotherapy, and 1 patient was treated with anti-oestrogen medication (1.5%).

## Discussion

The present study evaluated the frequency of ovarian metastases from nonovarian cancer in patients who underwent FDG PET/CT as part of the diagnostic workup for suspected ovarian cancer. In only 4% of this patient population, biopsy revealed ovarian metastases from nonovarian cancer; this is in line with the findings of Skírnisdóttir et al, who reported a frequency of 2.3% among 10 955 Swedish patients,^5^ and Antila et al, who reported a frequency of 5% among 725 Finnish patients.^3^ In both studies, the patients were identified retrospectively and had biopsy-verified nongenital tract metastases to the ovaries. However, the frequency of 4% is much lower than that reported by Yada-Hashimoto et al, who reported a frequency of 21% among 304 patients,^4^ and de Waal et al, who reported a frequency of 15% among 764 patients.^2^ However, these 2 studies also included metastases from the genital tract, including primary ovarian cancer metastasising to the contralateral ovary, which might partly explain the higher frequency.

Patients often present with symptoms such as abdominal distension, changes in stool, pain, or postmenopausal bleeding. Depending on these symptoms, they may be directed to different specialties, including Gynaecology or Gastrointestinal Surgery. Since 2006, the Department of Gynaecology has primarily used FDG PET/CT as the initial imaging modality when cancer was suspected giving rise to the population included in the present study. Another option for the evaluation of suspected ovarian cancer is MRI. However, it seems that both FDG PET/CT and MRI are inadequate for a precise diagnosis and staging of ovarian cancer and currently much research is devoted to the evaluation of new PET tracers, MRI sequences and other diagnostic procedures for the diagnosis of malignancy in the ovaries.^21–23^

In the present study, the most common cause of ovarian metastasis was lower gastrointestinal cancer, accounting for almost half of the cases, followed by nonovarian gynaecologic cancer and breast cancer. These findings are consistent with those from several studies that investigated the primary site of metastases to the ovaries.^2^^,^^5^^,^^6^^,^^10^^,^^24^ Consistently, all studies found that the most common origin of metastases to the ovaries was the gastrointestinal tract. The least common cancer that metastasizes to the ovaries is haematologic cancer. This low number might be understated, as increased ovarian FDG uptake in a patient with lymphoma is commonly evaluated by FDG PET/CT during treatment and is only biopsied if FDG uptake persists while the remaining lymphoma-related FDG uptake decreases during treatment.

In the present study, 40% of the included patients had a known diagnosis of nonovarian cancer. In this group of patients, ovarian metastases originated from known nonovarian cancer in more than 2 out of 3 patients. The mean time from the previous cancer diagnosis to the development of ovarian metastases was 4.3 years, ranging from 47 days to 11 years. Similar findings were shown by Zhang et al.^24^ They found that 46% of patients with ovarian metastases had known previous cancer, which was the cause of ovarian metastasis, and that the time from diagnosis to the presence of ovarian metastases ranged from 1.5 months to 20 years after cancer diagnosis. Furthermore, Zhang et al reported variations in the interval between metastases of different primary tumours, for example, metastasis from gastrointestinal cancer occurs at 1-2 years, and metastasis from the bladder, pancreas and appendix occurs approximately 10 years after the primary tumour; this is in line with the findings of this study, with metastases from lower gastrointestinal cancer occurring after approximately 2 years and metastases from cancer in the urinary tract occurring after approximately 5 years. Additionally, we found that patients with metastases from breast cancer had the longest time to metastasis, at 7 years.

It is important to correctly diagnose patients to optimize treatment and thereby hopefully increase survival rates. Imaging is non-invasive and could potentially contribute to distinguishing between primary ovarian cancer and metastases to the ovaries, as the prognosis is worse for patients with ovarian metastasis, compared to primary ovarian cancer,^5–7^ as this reflects an advanced stage of the cancer disease. However, it is worth noting that the prognosis depends on the primary tumour; for instance, the prognosis of patients with metastases to the ovaries originating from the genital tract is better than that of patients with ovarian metastasis originating from the pancreas or small intestine.^2^^,^^7^

Previous studies have reported that patients with ovarian metastases tend to be younger than patients diagnosed with primary ovarian cancer^5^^,^^7^^,^^24^; for example, Zhang et al reported a mean age of 48 years for patients with ovarian metastases. However, in agreement with Antila et al, our own results did not confirm this.^3^ The mean age at diagnosis in this study was 59 years, which is similar to the age of patients with primary ovarian cancer who are typically diagnosed in their late 50s to early 60s.^25^ It is also important to note the wide age range at diagnosis and that the diagnosis is not limited to a certain age range.

To distinguish between ovarian metastasis and primary ovarian cancer, several studies have investigated tumour size and characteristics. Generally, metastases are smaller (<10 cm) and are more often bilateral than primary ovarian cancer.^2^^,^^3^^,^^7^ Kubeček et al noted that metastases from breast cancer are usually smaller, and metastases from the gastrointestinal tract tend to be larger and present more like primary ovarian cancer.

The majority of patients in this study with available CA-125 had elevated levels, but CA-125 does not seem to be a helpful biomarker for distinguishing metastases to the ovaries from primary ovarian cancer,^3^^,^^7^ as elevated levels can be found in 80% of women with primary epithelial ovarian cancer^26^ and in 70% of women with ovarian metastasis^7^; however, Zhang et al suggested that increases in CA-125 levels of up to several thousand times are relatively common in patients with epithelial ovarian cancer and not in those with metastases to the ovaries, where the CA-125 level does not increase to the same level.^24^ Other papers suggest including other biomarkers, such as CA-19-9 and carcinoembryonic antigen, to separate primary ovarian cancer from gastrointestinal cancer or pancreatic cancer in the investigation of patients with unknown ovarian masses,^2^^,^^3^^,^^24^ especially if they have a previous history of malignant disease, as we found that metastasis to the ovary can occur up to 11 years after the original cancer diagnosis.

In the original evaluations of FDG PET/CT scans, more than half of the evaluations suggested primary ovarian cancer. However, only 17% of the evaluations suggested metastases to the ovary from nonovarian cancer; this is likely due to the fact that metastases mimic primary ovarian cancer, for example, size, or because they present years after the treatment of the primary cancer as ovarian metastases without signs of local recurrence of the primary cancer.

The median SUV_max_ of the ovarian metastasis in this study was 5.39 (range 0.75-26.69), which is in accordance with the findings of Lee et al and Kitajima et al, who reported SUV_max_ values of 5.3 ± 2.6 and 4.6 ± 2.4, respectively.^10^^,^^11^ Furthermore, both studies reported higher SUV_max_ values in metastases from breast cancer and colon cancer, which correlates with the measured SUV_max_ values of this study and with the results from Park et al.^8^ Multiple studies have investigated the SUV_max_ of primary ovarian cancer, with varying results ranging from 5.31 to 10.5.^9^^,^^10^^,^^12^ Furthermore, Lee et al reported a significant difference between the SUV_max_ of metastases to the ovaries and that of primary ovarian cancer, yet the ranges overlapped to a large extent, making it difficult to differentiate the groups. The median SUV_max_ of this study was 5.39, which could suggest that the SUV_max_ of metastasis is lower than that of primary ovarian cancer but also that the SUV_max_ alone is not sufficient to differentiate between the 2.

Limitations of FDG PET/CT include the inability to detect small lesions (<1.0 cm) and tumours without or with low FDG uptake (eg, lobular breast cancer, renal cell carcinoma and some GI cancers).^7^^,^^12^

The ability of FDG PET/CT to distinguish ovarian metastases from primary ovarian tumours is debated. Kubeček et al questioned this ability, whereas Park et al emphasized that FDG PET/CT is vital for the early detection of metastatic tumours, regardless of whether they are primary or metastatic tumours, as it is important for treatment planning.^7^^,^^8^ Furthermore, when using SUV values, it is important to consider the scan and reconstruction protocol at the scan facility, as the time from FDG injection to scan and blood glucose levels may affect the SUV values.

In premenopausal women, incidental FDG uptake in the ovaries is common because it is related to the menstrual cycle and rarely indicates malignancy.^8^^,^^27^^,^^28^ However, Zhang et al noted that increased blood flow to the ovaries is considered a contributing factor for ovarian metastasis, making it an important differential diagnosis in premenopausal women.^24^ However, in postmenopausal women, increased FDG uptake usually suggests malignancy.^8^^,^^28^

While our retrospective study provides valuable insights into metastases to the ovaries in a Danish population with equal rights to public health care, it is important to acknowledge the limitations that may affect the interpretation and generalizability of our findings. The patients included in this study were limited to patients with FDG PET/CT and biopsy-verified metastasis. While this factor contributes to the reliability of the study, the inclusion criteria may have introduced selection bias. Furthermore, the patients were selected from a group of patients suspected of having ovarian cancer, and even more patients could be included if all FDG PET/CT scans from the time period were included.

The completeness and accuracy of the data relied on the records available in the electronic medical records, which in Denmark is linked to a unique social security number making follow-up more complete. However, incomplete or missing data for certain variables may have influenced the results. Furthermore, unmeasured or inadequately controlled confounders may have impacted the results. Caution should be exercised when generalizing the findings to populations with different demographic or clinical characteristics, as our study population represents a relatively small sample size and may not be fully representative of the broader population of patients with ovarian metastases.

In conclusion, in an unselected population evaluated with FDG PET/CT due to suspicion of ovarian cancer, the frequency of metastases to the ovary from a nonovarian cancer was 4%, and the most common origin of ovarian metastasis was the lower gastrointestinal tract. A previous cancer history is an important factor to consider when assessing an unknown tumour of the ovary, as metastasis can occur many years after ending treatment for a previous cancer. The SUV_max_, age, tumour size, and CA-125 concentration are not clinically useful markers for distinguishing primary ovarian cancer from ovarian metastases.
